# Handheld Photoacoustic Microscopy Probe

**DOI:** 10.1038/s41598-017-13224-3

**Published:** 2017-10-17

**Authors:** Kyungjin Park, Jin Young Kim, Changho Lee, Seungwan Jeon, Geunbae Lim, Chulhong Kim

**Affiliations:** 1 School of Interdisciplinary Bioscience and Bioengineering, Pohang University of Science and Technology (POSTECH), 77 Cheongam-ro, Nam-gu, Pohang, Gyeongbuk 37673 Republic of Korea; 2 Department of Creative IT Engineering, Pohang University of Science and Technology (POSTECH), 77 Cheongam-ro, Nam-gu, Pohang, Gyeongbuk 37673 Republic of Korea; 3 Department of Mechanical Engineering, Pohang University of Science and Technology (POSTECH), 77 Cheongam-ro, Nam-gu, Pohang, Gyeongbuk 37673 Republic of Korea; 4 Present Address: Department of Nuclear medicine, Chonnam National University Medical School & Hwasun Hospital, 160 Baekseo-ro, Gwangju, 61469 South Korea

## Abstract

Optical resolution photoacoustic microscopy (OR-PAM) is a non-invasive, label-free method of *in vivo* imaging with microscopic resolution and high optical contrast. Based on intrinsic contrasts, OR-PAM has expanded to include *in vivo* vessel imaging, flow cytometry, physiological parameter analysis, and single-cell characterization. However, since conventional OR-PAM systems have a fixed tabletop configuration, a large system size, and slow imaging speed, their use in preclinical and clinical studies remains limited. In this study, using microelectromechanical systems (MEMS) technology, we developed a handheld PAM probe with a high signal-to-noise ratio and image rate. To enable broader application of the OR-PAM system, we reduced its size and combined its fast scanning capabilities into a small handheld probe that uses a 2-axis waterproof MEMS scanner (2A-WP-MEMS scanner). All acoustical, optical, and mechanical components are integrated into a single probe with a diameter of 17 mm and a weight of 162 g. This study shows phantom and *in vivo* images of various samples acquired with the probe, including carbon fibers, electrospun microfibers, and the ear, iris, and brain of a living mouse. In particular, this study investigated the possibility of clinical applications for melanoma diagnosis by imaging the boundaries and morphology of a human mole.

## Introduction

Optical-resolution photoacoustic microscopy (OR-PAM) is a non-invasive, label-free, *in vivo*, volumetric imaging modality with microscopic resolution and high optical contrast^1,2^. In OR-PAM, the optical excitation light is tightly focused, and thus high resolution can be achieved in the quasi-ballistic regime. The focused light is absorbed by endogenous chromophores, such as two types of hemoglobin, melanin, DNA/RNA, water, and lipid. The consequent heat generation through optical absorption causes the emission of acoustic waves as a result of thermoelastic expansion. The generated acoustic waves propagate in the medium and are detected by an ultrasound transducer. The time-differentiable photoacoustic (PA) signals are translated into images that reveal structural information, such as microvasculature^2,3^ and red blood cells^4^, and functional information such as total hemoglobin concentration (*C*_Hb_)^2^, hemoglobin oxygen saturation (sO_2_)^5^, blood flow^6^, and the metabolic rate of oxygen consumption^7^. The signals also provide molecular-level information, including intravital imaging of amyloid plaques^8^, malignant cells (with the use of targeted contrast agents)^9–11^, and imaging of fluorescent proteins^12–14^.

Exploiting OR-PAM’s versatility, researchers have extensively applied it in cell and live animal studies. The subjects of these studies have included the detection of melanosomes in melanoma cells^15^, flow cytometry for the detection of the circulating tumor cells (CTCs)^16,17^, tumor angiogenesis^18^, label-free histological imaging of DNA and RNA^19^, single cell flowoxigraphy^20–23^, ocular angiography^24^, and monitoring neovascularization^25,26^. In addition, OR-PAM has imaged the microvasculature of the human finger cuticle and the hemodynamic parameters in a cuticle capillary loop, including *C*_Hb_, sO_2_, and blood flow rate^27^.

The first commercialized OR-PAM system in a table-top configuration was equipped with an opto-ultrasound combiner (OUC) for confocal and co-axial alignment of both light and ultrasound to achieve a high signal-to-noise ratio (SNR) and high spatial resolution^28^. The OUC enables the signal maximized by making the dual foci of the light illumination and ultrasound detection. The lateral resolution of the system is also enhanced as the focus of the light becomes tighter because the focus of the light is much smaller than the ultrasound in the dual foci. However, this system used a mechanical raster scanning mechanism with a 2-axis-motorized stage. Thus, the system was bulky and suffered from an inherently slow imaging speed, limiting its widespread use in both preclinical and clinical studies. As with conventional ultrasound imaging, deploying this technology in a variety of applications requires the development of a real-time, portable OR-PAM imaging system. Seeking a handheld tool, in 2011 Hajireza *et al*. developed a fiber bundle-based handheld OR-PAM with a compact footprint of 4 cm by 6 cm^29^. However, its slow imaging speed and small field of view (FOV) (400 μm × 400 μm), caused by its motorized stage and the absence of an opto-ultrasound combiner, limited the wide application of the system. A high-resolution linear-array-based combined PA and US system, such as the Vevo LAZR from Fujifilm VisualSonics, offers improved imaging speed and depth for imaging the human microcirculation and providing functional information^30–32^. However, the system resolution is determined by ultrasound beamforming instead of optical focusing. Thus, it is difficult to achieve resolutions finer than 10 micrometers, despite the use of a high-frequency transducer.

In this study, we demonstrate a high SNR and high speed handheld photoacoustic microscopy (PAM) probe that uses microelectromechanical systems (MEMS) technology^33,34^. Most notably, this handheld system integrates all the acoustical, mechanical, and optical components into a single probe with a diameter of 17 mm and a weight of 162 g. A PA image with 700 × 700 pixels is obtained, with measured B-scan and volumetric imaging speeds of 35 Hz and 0.05 Hz, and the laser is operated at 532 nm with a 50-kHz-repetition rate. The measured lateral and axial resolutions are 12 μm and 30 μm, respectively. Finally, we demonstrate phantom and *in vivo* images of various samples, including carbon fibers, electrospun microfibers, and live animals (e.g., a mouse ear, iris, and brain). Of particular interest, we use the system to delineate a human mole, a step toward immediate clinical application in delineating melanomas. Melanoma is only 1% of all skin cancer cases, but has the highest death rate among them; in 2017, approximately 9,730 deaths are predicted in the United States^35^. Overexposure to ultraviolet light (UV) causes melanocytes, pigment-containing skin cells, to develop a malignant melanoma. Because atypical human moles are precursors of melanomas, we investigated the possibility of clinical applications for melanoma diagnosis by imaging the boundaries and morphology of a mole on a human subject.

## Results

### Structure of the two-axis water-proof MEMS scanner

**Figure 1:**
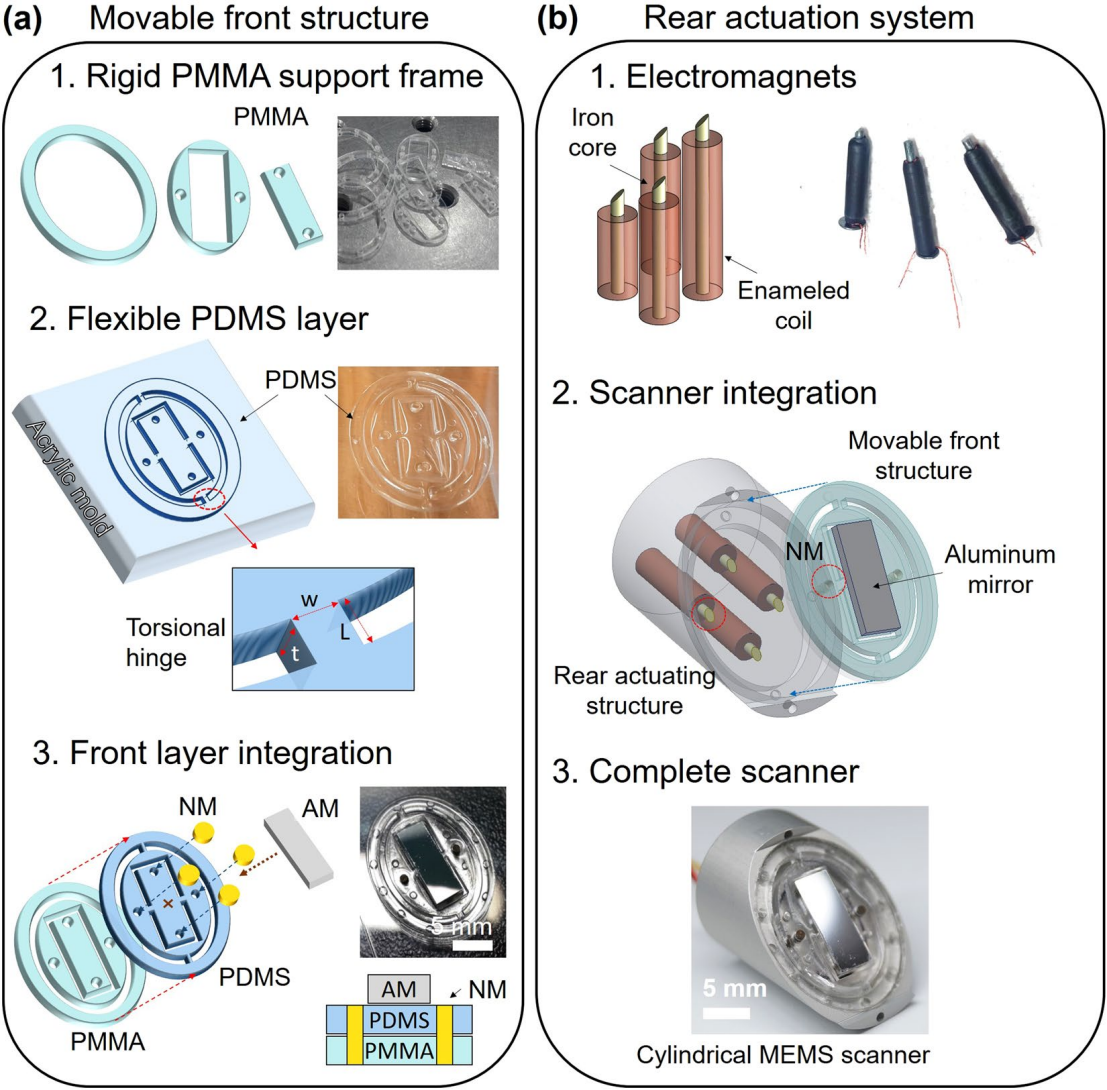
Structure of the two-axis water-proof MEMS scanner. Fabrication processes of (**a**) the movable front structure and, (**b**) the rear actuation system. PDMS, Polydimethylsiloxane; PMMA, Poly methyl methacrylate; NM, neodymium magnet; AM, aluminum mirror; EM, electromagnets.

**Figure 2:**
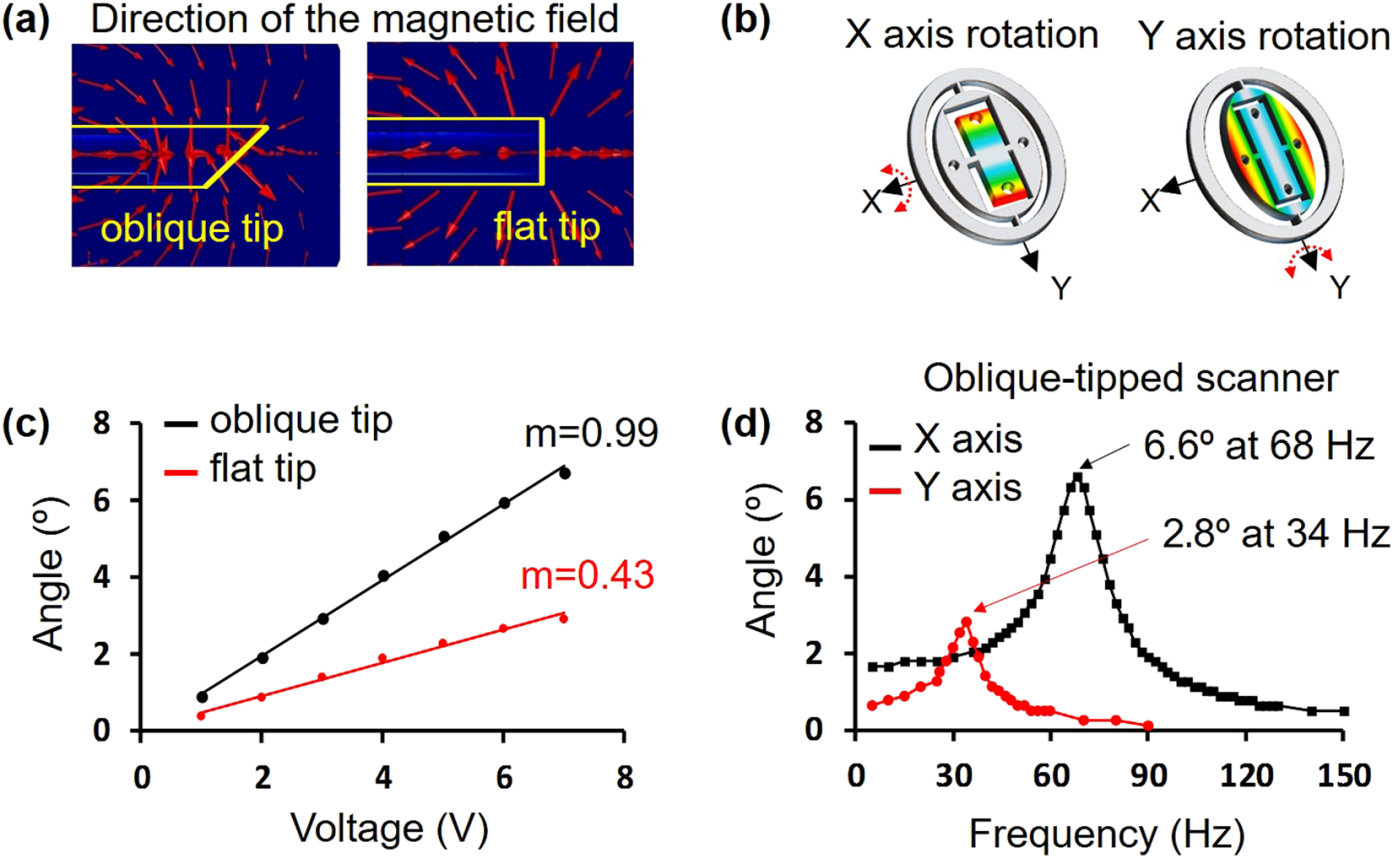
Simulation and scanning responses of the 2A-WP-MEMS scanner. Representations of the magnetic fields from (**a**) an oblique-tip electromagnet and (**b**) a flat-tip electromagnet. (**c**) Rotational movement of the movable front structure along the x axis. (**d**) Rotational movement of the movable front structure along the y axis. (**e**) Scanning angles versus the applied DC voltages along the x axis. m, slope of the line. (**f**) Scanning angles versus applied AC frequencies at 2 V along the x and y axes; color in C and D indicates the displacement from the original position.

**Figure 3:**
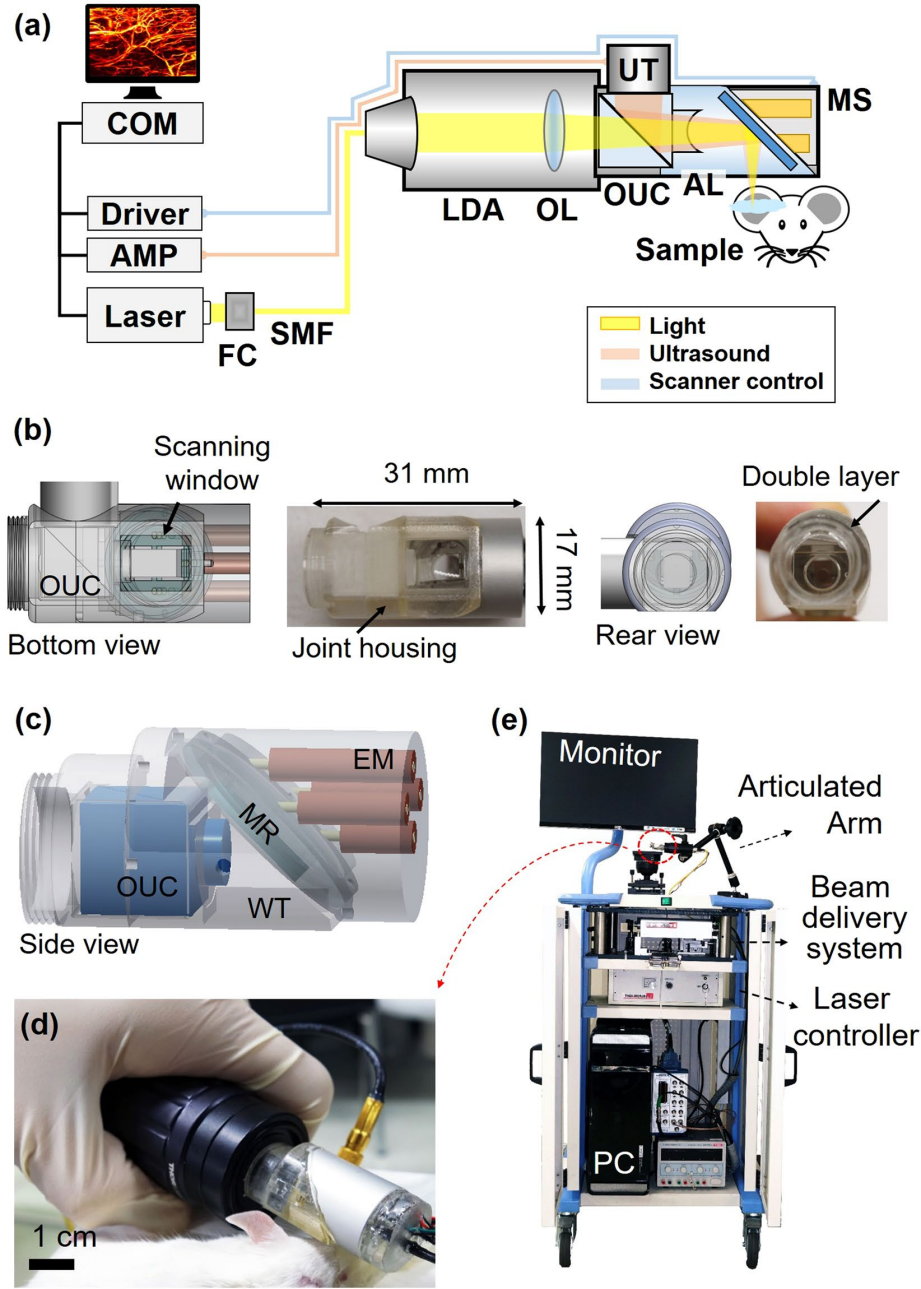
Handheld photoacoustic microscopy (PAM) probe. (**a**) Schematic of the whole handheld PAM probe system. (**b**) Bottom and rear views of the probe: CAD design and photograph. (**c**) 3D schematic of the probe. (**d**) Photograph of the handheld PAM probe. (**e**) Photograph of the entire system in a medical cart. AL, acoustic lens; AMP, amplifier; AT, adjustable tube; CL, collimation lens; COM, computer; EM, electromagnets; FC, fiber collimator; LDA, light delivery assembly; MR, mirror; MS, MEMS scanner; OL, objective lens; OUC, opto-ultrasound combiner; SMF, single mode fiber; UT, ultrasonic transducer; WT, water tank.

The new two-axis waterproof MEMS scanner (2A-WP-MEMS scanner) consists of a movable front structure with a reflecting mirror, and a rear actuating structure that pivots the reflector along the two axes. The movable front structure steers the light and ultrasound at the same time. It consists of three separate rigid PMMA (methyl methacrylate) supporting elements, a flexible PDMS (polydimethylsiloxane) layer, four neodymium magnets (NM), and a light-and-ultrasound reflecting aluminum mirror (AM) (Fig. 1a). The bottom layer of the movable front structure consists of the rigid PMMA support pieces, which provide stiffness (Fig. 1a-1). The flexible layer in the movable front structure is made of PDMS. (Fig. 1a-2), a polymeric organosilicon compound that is nonconductive and waterproof for working in water. The structural difference between the bottom supports and the flexible PDMS layer is the four torsional hinges on the flexible PDMS layer, which lie atop voids in the bottom PMMA supports. Three separate PMMA frames rigidly support the flexible PDMS layer, and each part has multiple alignment marks to help precisely align the PDMS layer with the bottom PMMA support. After the two layers are glued together, four neodymium magnets are placed in the middle of the PDMS-PMMA structure: two in the central moving part, and two in the outer moving parts (Fig. 1a-3). On top of the PDMS layer, the aluminum mirror is attached at the center of the movable structure. Reflectivity can vary with angle, but generally at normal incidence the aluminum mirror reflects both light, with a reflection rate of 92%, and ultrasound, with a reflection rate of 84% in water^34^. In this configuration, all four parts are combined into one movable front structure (Fig. 1a-3). The rear actuating structure generates torsional force to pivot the movable front structure on two axes (Fig. 1b). To generate the magnetic field that provides the torsion, we hand-crafted four electromagnets (Fig. 1b-1) that fit into holes in the aluminum housing (Fig. 1b-2), spaced an equal distance apart in a diamond. The distance is optimized to decouple the electromagnetic force on each axis. In use, the aluminum housing dissipates heat released by the electromagnets, helping to suppress resistance change. The whole rear actuating structure is sealed with PDMS for electrical insulation. The moving front structure is combined with the rear actuating structure as shown in Fig. 1b-3. The front structure, including the light-reflecting aluminum mirror, is tilted 45° with respect to the long axis of the scanner. The four magnets on the front structure are directly aligned with the tips of the four electromagnets in the rear actuating structure (red circle in Fig. 1b-2), using the alignment marks on both the aluminum body and the back side of the PMMA support frames.

### Numerical analysis and scanning responses of the 2A-WP-MEMS scanner

Unlike our previous rectangular MEMS scanner^33^, the new 2A-WP-MEMS scanner is an obliquely truncated cylinder, which reduces the diameter of the probe. Because the shape of the scanner has been changed, the previous tip geometry of the electromagnets has to be modified and the neodymium magnets in the movable front structure have to be repositioned. To determine the optimal tip shape of the electromagnet, we numerically analyzed a flat tip and an oblique tip electromagnet (Fig. 2a). The simulation results show that an oblique tip electromagnet provides a magnetic field that is better aligned with the permanent magnets in the inclined front structure. Guided by the simulation results, we machined the tips of the four electromagnet cores at an oblique angle to actuate the correspondingly angled front layer, and positioned the four neodymium magnets perpendicular to the oblique tip surfaces. Following up on the magnetic field simulation and fabrication, we experimentally tested the scanning responses of the new 2A-WP-MEMS scanner (Fig. 2b). The scanning angles were measured by applying DC voltage to both oblique and flat tip electromagnets. The magnetic forces generated by both oblique and flat tipped electromagnets were linearly proportional to the applied DC voltages (Fig. 2c), as expected from Eq. (1).

1 $${\boldsymbol{B}}=\widehat{{a}_{z}}\frac{{\mu }_{0}NI\,{b}^{2}\,}{2\,{({z}^{2}+{b}^{2})}^{3/2}}$$

Where ***B*** is the magnetic field, $\widehat{{a}_{z}}$ is the unit vector directed from the source point to the field point in the vertical direction, *μ*_0_ is the permeability of free space, N is the number of turns wound on the electromagnet, *I* is the current through the solenoid coil, *b* is the radius of the coil, and *z* is the gap between the solenoid coil and the magnet. In the graph of scanning angle versus applied DC voltage, the slope of the line from the flat tip electromagnet is 0.43, whereas that from the oblique tip electromagnet is 0.99 (Fig. 2c). This experimental result shows the oblique tip electromagnet provides double the force of the flat tip electromagnet. Another important characteristic of the MEMS scanner actuation is its resonant frequency. By operating the MEMS scanner at the resonant frequency, the maximum FOV can be obtained at a low voltage. By applying AC voltage on both the x and y axes of the oblique tip scanner, we measured the scanning angles to obtain the resonant frequency (Fig. 2d). The maximum displacement angle of the scanner with respect to the x axis was 6.6°, at a resonant frequency of 68 Hz, and the maximum displacement angle of the scanner with respect to the y axis was 2.8°, at a resonant frequency of 34 Hz (Fig. 2d).

### Handheld photoacoustic microscopy probe

After simulating, fabricating, and testing the new 2A-WP MEMS scanner, it was integrated into the PAM probe. The major challenge was to integrate the optical and acoustical guidance systems with the mechanical scanning subsystems in a compact probe (Fig. 3a). During the integration, the SNR was a key factor that allowed us to avoid averaging multiple frames. To maximize the SNR, the OUC and the aluminum mirror on the MEMS scanner were used for confocal and co-axial alignment of the light and ultrasound. Further, an acoustic lens with a numerical aperture of 0.25 was mounted in front of the OUC. However, due to the short 11 mm acoustic focal length, a complicated housing structure was needed to accommodate the MEMS scanner in the acoustic focal path. The housing that connects the MEMS scanner, OUC, transducer, and a light delivery assembly was fabricated by 3D printing, using a transparent photo polymer (Fig. 3b–c). On the rear side of the housing, a light delivery assembly (LDA) was attached (Fig. 3a). The scanning window at the bottom of the probe was encapsulated by a thin polyethylene film. This waterproof sealing film also acted as an impedance matching layer that was transparent to both light and ultrasound (Fig. 3c). The assembled probe weighed 162 g, its diameter was 17 mm, its length is 31 mm, the diameter of the LDA was 30 mm, and the total length of the whole probe including light delivery was 12 cm (Fig. 3d). The completed handheld PAM probe was mounted on a convenient and portable 3-axis articulated arm. The entire system, including the laser delivery system, the data acquisition & signal processing system, and the handheld probe, was placed on a medical cart for portability (Fig. 3e).

### Spatial resolutions, SNR, and linearity of the handheld PAM probe

**Figure 4:**
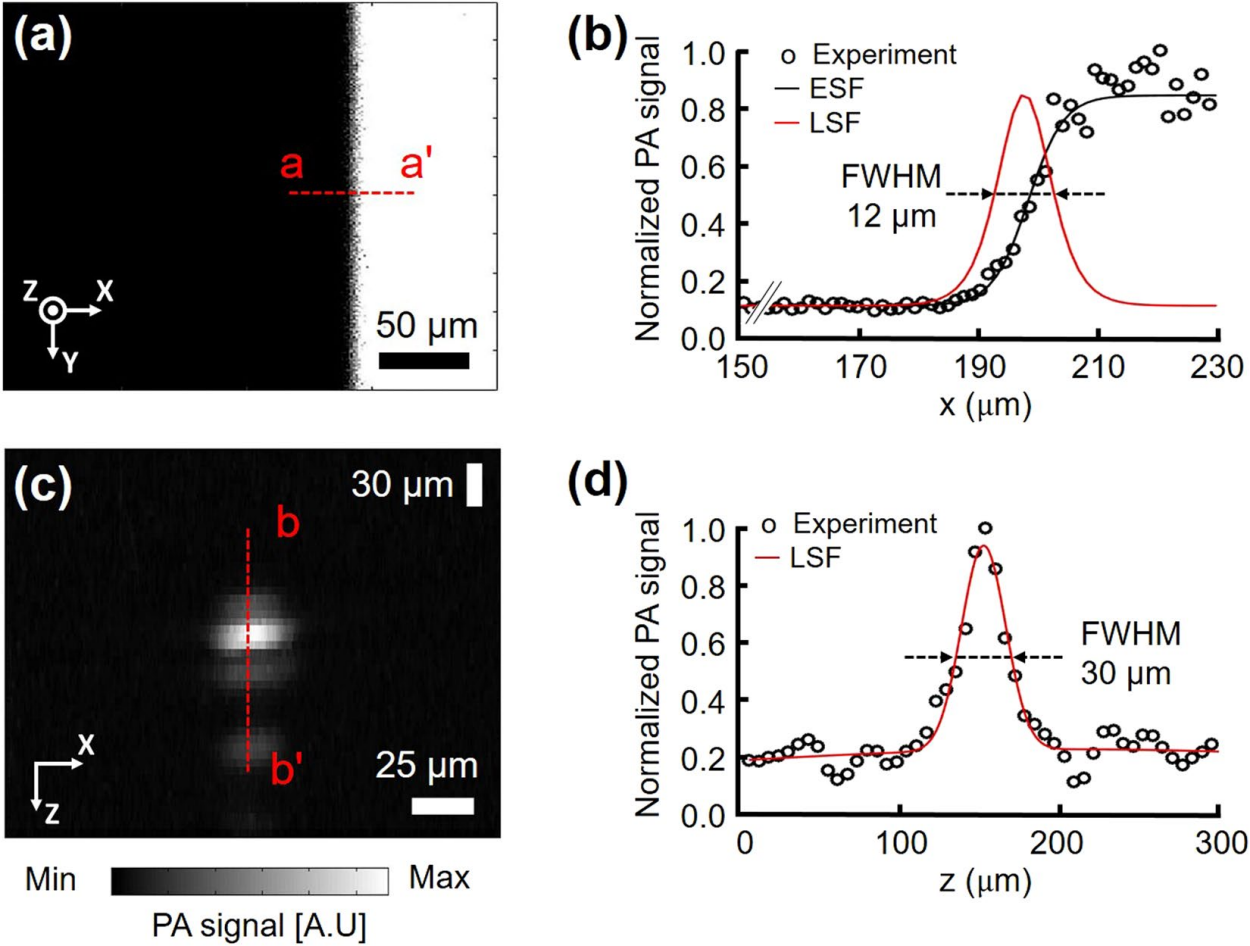
Spatial resolutions of the handheld PAM probe. (**a**) PA MAP image of part of a black and white pattern. (**b**) LSF fitting from the experimental data across the line a-a’ in (**a**). (**c**) Cross-sectional PA B-scan image of a carbon fiber. (**d**) LSF fitting from the experimental data across the line b-b’ in (**b**). FWHM, full width at half maximum; MAP, maximum amplitude projection; ESF, edge spread function; and LSF, line spread function.

After building the system, we measured its spatial resolution. To measure its lateral resolution, we prepared a micro-patterned black film mask and obtained a PA maximum amplitude projection (MAP) image (Fig. 4a). From this image, B scan profile data for one scan were extracted across the x direction along the line a-a’. The measured lateral resolution from the micro patterned black film was 12 ± 1.6 μm, while the theoretical value is 4.5 μm (Fig. 4b). One possible cause of the difference is the aberration at the boundary of the acoustic lens. This optical system needs a correction lens on the OUC at the light-receiving side to reduce aberration. However, because of the short working distance of the acoustic lens, we could not attach a correction lens. The spatial resolution should be defined in terms of the ability of an imaging system to resolve two closely spaced objects. To verify the measured system resolution, we imaged the patterns of group 6 in the USAF resolution test chart (Fig. S2). The single line width and the gap between lines of the third element in group 6 are 6.2 μm. The third element and even smaller patterns (5.5 μm for the forth element, 4.9 μm for the fifth element) are also identified. Therefore, the system resolution is better than the measured resolution of 12 μm obtained above. After the lateral resolution measurement, we captured a PA B-scan (i.e., a depth-resolved two dimensional image) image of a 6-μm diameter carbon fiber to measure the axial resolution (Fig. 4c), and extracted the raw PA data across the fiber in the depth direction. This extracted PA signal was normalized to a maximum value of one, then fitted to a line spread function (LSF). The FWHM (FWHM) was obtained from this Gaussian profile. The measured axial resolution was 30 ± 3.1 μm, while the theoretical value is 27 μm, $({R}_{A,OR/AR}=0.88{\nu }_{A}/{\rm{\Delta }}{f}_{A})$ (Fig. 4d). In addition, a PA image of the 6-μm diameter carbon fiber was used for the SNR measurement. The measured SNR of our PAM probe was 39.2 dB at 532 nm, which is approximately 0.84 times less than that of a second-generation OR-PAM^2^ (42.7 dB at 570 nm). One possible reason could be the wavelength difference between the two systems. For instance, the absorption coefficient of blood is 0.96 times smaller at 532 nm than at 570 nm. Thus, our system’s sensitivity could be comparable with the G2-OR-PAM’s sensitivity^2^. Furthermore, we imaged numbers on a micro-ruler to check the linearity of the system in the specific FOV of 1 mm by 1 mm, and 2 mm by 2 mm (Fig. S3). In summary, by measuring the spatial resolution and the SNR of the system, we can conclude that this system can detect microvasculature in a live animal without averaging multiple frames of image.

### PA imaging of electrospun fibers

**Figure 5:**
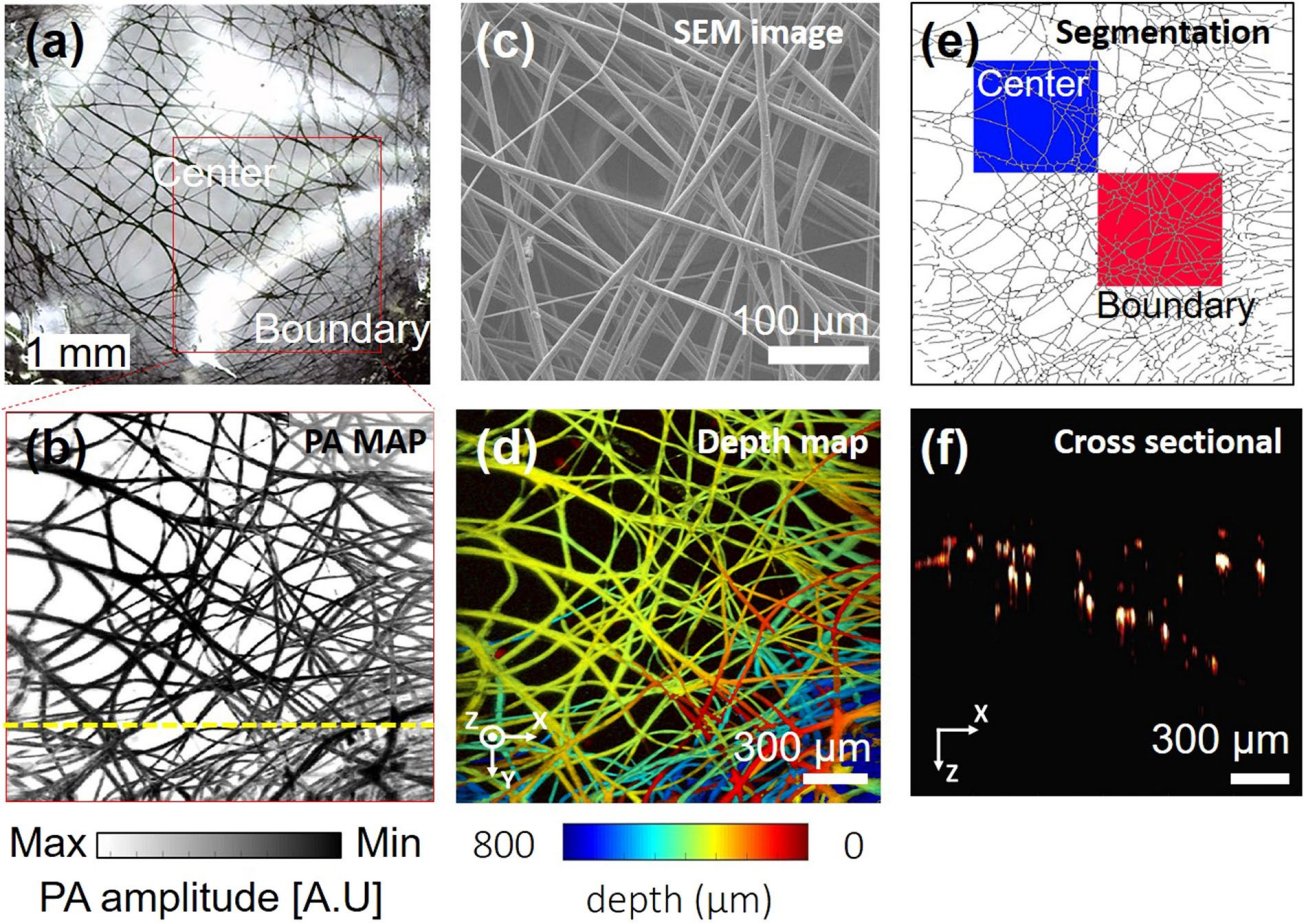
PA image of electrospun microfibers. (**a**) Photograph of electrospun microfibers dyed with black ink. (**b**) SEM image of electrospun microfibers. (**c**) PA MAP image of electrospun microfibers. (**d**) Depth map image corresponding to (**c**); pseudo color encodes depth information. (**e**) Computed-thinned image of electrospun microfibers. Blue box indicates a sampling area in the center region; red box indicates a sampling area near the boundary region. (**f**) Cross-sectional B-scan image of electrospun microfibers along the yellow dotted line in (**b**). SEM, scanning electron microscopy and MAP, maximum amplitude projection.

After measuring the spatial resolution and the SNR, we next demonstrated the system’s ability to image microvasculature. We photoacoustically imaged a vessel-mimicking phantom composed of a membrane of black-dyed electrospun polycaprolactone (PCL) microfibers (Fig. 5a–b). As measured by a scanning electron microscope (SEM), each fiber’s diameter is about 10 μm, (Fig. 5c), similar to that of the tiniest blood vessels in the human body. A PA MAP image was obtained from the PCL membrane area within the red box in Fig. 5a. From the obtained PA MAP image, we could observe the morphology of the fiber threads, clearly identify single fibers, and visualize multiple overlapped thread regions. However, some apparently overlapping fibers in the image are not physically contiguous, but are simply imaged at different depths. To distinguish threads at different depths, the PA MAP image was converted to a depth map image (Fig. 5d). In the depth map image, the depth information is translated to RGB color, which shows threads at different depths in the boundary region in multiple colors, whereas threads in the center that are physically overlapped appear in monocolor. Furthermore, the depth map image describes the fiber density gradient of the membrane. The fiber threads are sparse at the center of the membrane and dense at the edge. To quantify the density of the electrospun microfibers, we segmented each fibers (Fig. 5e). Two square boxes with identical dimensions of 0.67 mm by 0.67 mm were designated to calculate the total fiber length per unit area for density quantification. The blue box encloses an area near the center, and the red box indicates an area near the boundary. The calculated total length of the fibers in the blue box was 21 mm, and the total fiber length in the red box was 31 mm (Fig. S4). By calculation, we could conclude that fibers in the center of the membrane are two-thirds sparser than at the boundary. The cross-sectional image (Fig. 5f) along the yellow dotted line shown in the Fig. 5b is well-matched with the expected surface morphology from the depth map image of the Fig. 5d. The imaging acquisition and display time for 700 × 700 pixels along the x and y axes was 20 s, with a FOV of 2 mm by 2 mm. By imaging a micro vessel mimicking phantom, we showed that the system was capable of imaging microvasculature in a live animal, and that the errors caused by the MEMS scanner’s scanning profile (Fig. S6) could be corrected.

### *In vivo* PA images of a mouse ear, iris, and brain microvasculature

**Figure 6:**
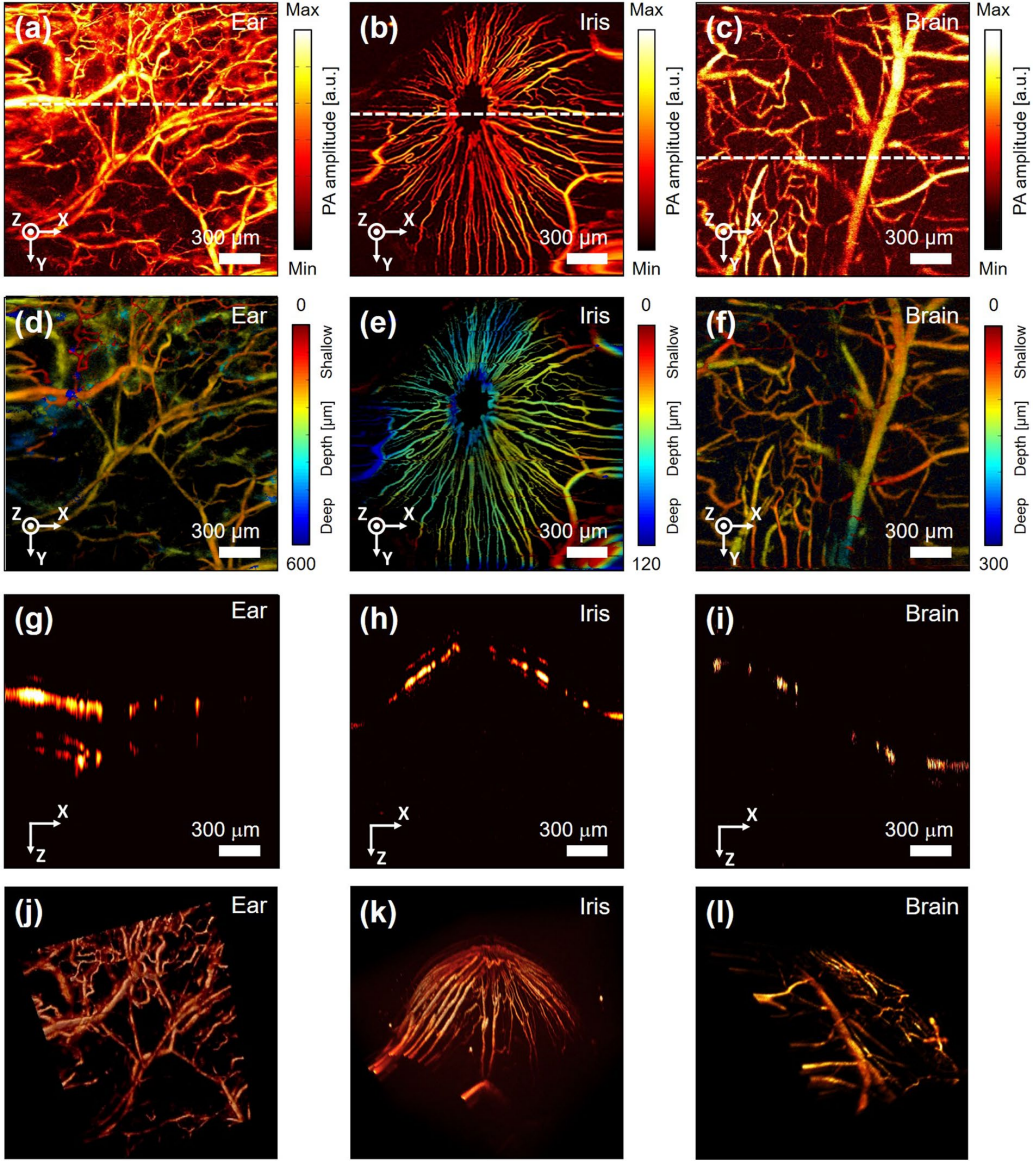
*In vivo* PA images of microvasculature in a mouse ear, iris, and brain (**a**), (**b**), (**c**) PA MAP images of microvasculature in a mouse ear, iris, and brain. (**d**), (**e**), (**f**) Depth map image of top views of microvasculature in the mouse ear and brain, and a frontal view of the microvasculature in a mouse iris. Pseudo color indicates depth information. (**g**), (**h**), (**i**) B-scan cross sectional images of the mouse ear, iris, and brain along the white dashed lines in (**d**), (**e**), and (**f**). (**j**), (**k**), (**l**) 3D PA volumetric images of a mouse ear (Supplementary Video S2), iris, and brain.

As an *in vivo* demonstration, we imaged the microvasculature in a mouse ear, iris, and brain, which were all clearly delineated in PA MAP images (Fig. 6a–c). The image of the vasculature in the ear (Fig. S5a) shows both capillary beds and single capillaries, as well as artery and vein pairs (Fig. 6a). The MAP image of the mouse iris shows the iris microvasculature (Fig. 6b). The image FOV of 2 mm by 2 mm was sufficient to capture the whole iris’s morphology in one frame. The MAP image of the mouse brain (Fig. S5b) shows brain cortical vessels (Fig. 6c). Unlike in the previous two images, it is difficult to find a capillary bed or single capillary. One possible reason is that the skull can hinder focusing the light tightly enough to resolve a capillary size blood vessel. In the depth map image of the mouse ear, the relative depth of each blood vessel is distinguishable (Fig. 6d). The depth map image of the microvasculature in the mouse iris shows a depressive morphology of the blood vessels at the center of the iris around the pupil (Fig. 6e). At the center of the iris around the pupil region, capillary loops are presented as continuous lines, indicating the circulation of the blood stream. The depth map image of the microvasculature in the mouse brain shows the cerebral circulation (Fig. 6f). The large blood vessels are thought to be veins because they are located on the surface near the cortex^36^. The image shows capillaries flowing around these large vessels. Cross-sectional B-scan images of the mouse ear, iris, and brain are shown in Fig. 6g–i, respectively. In the cross-sectional image of blood vessels in the mouse ear, we can clearly identify the flat morphology of the microvasculature surface (Fig. 6g). In the cross sectional image of the blood vessels in the iris, we can observe a curved elliptic cone shape (Fig. 6h). In the cross sectional image of the mouse brain, the crooked vasculature geometry is presented (Fig. 6i). *In vivo* PA imaging movie of the mouse ear from the LabView interface is attached to Supplementary video S1, and the total time to image 700 × 700 pixel was 20 seconds. The vasculature image of the mouse ear is rendered in 3D in Fig. 6j–l, and in Supplementary video S2. *In-vivo* experiments demonstrated the system can achieve the same PA images as the morphology of the vessels in real (Fig. S5).

### Extension of the FOV

**Figure 7:**
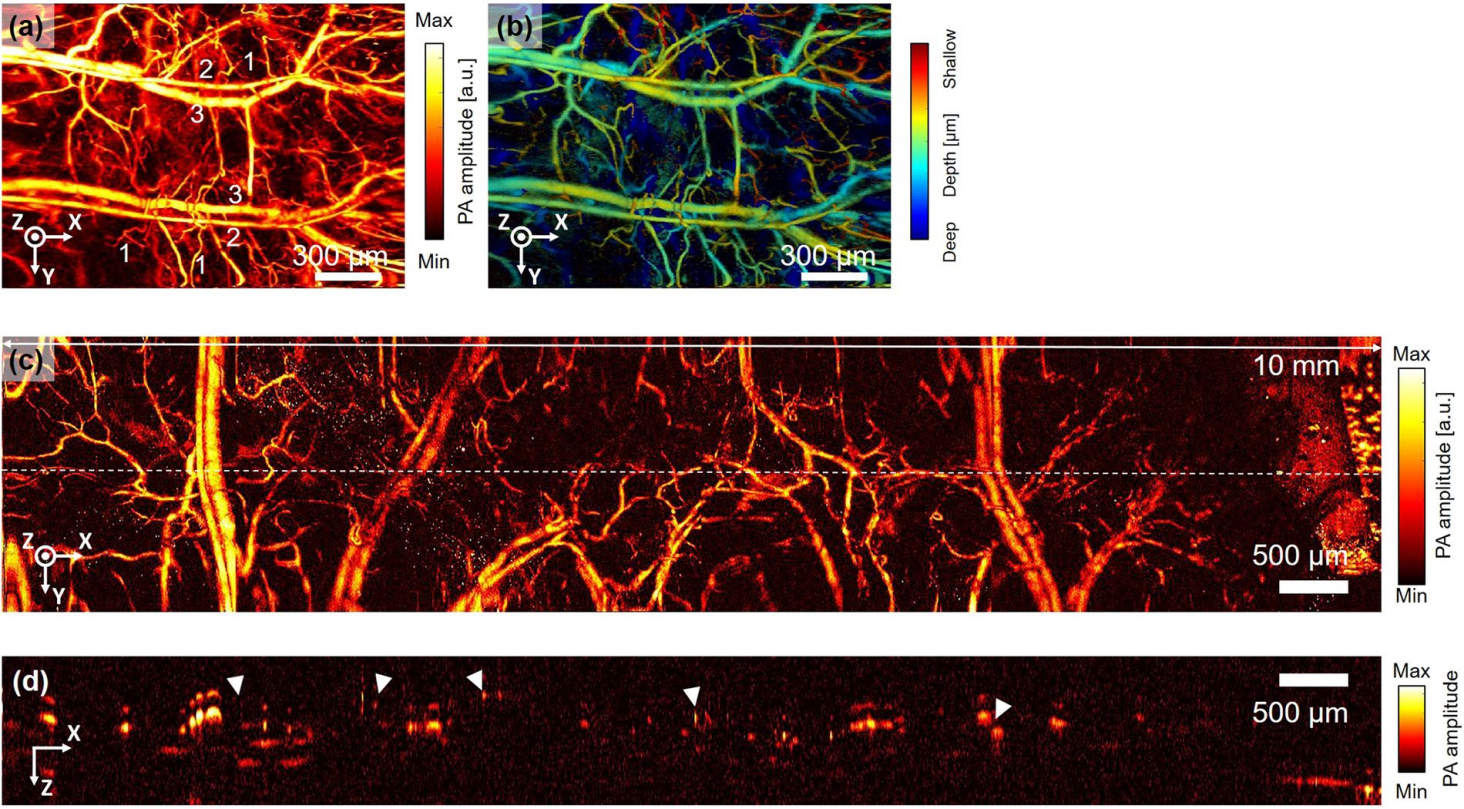
Extension of FOV. (**a**) Single PA MAP image of a mouse ear with an extended FOV (2.8 mm by 2 mm). (**b**) Depth map image corresponding to PA MAP image (**a**); pseudo color encodes depth information. (**c**) Mosaic PA MAP images made from eight PA MAP images (FOV 10 mm by 2 mm).

For clinical applications, a large FOV is required to image a wide range of biological tissue. To achieve a large FOV image with the current handheld PAM probe, we first experimentally increased the voltages to the electromagnets driving the MEMS scanner. In this experiment 3 V was applied on the x axis and 2 V was applied on y axis. As a result of increasing the x-axis voltage, a PA MAP image (Fig. 7a) of 2.8 mm by 2 mm was obtained from a mouse ear (Fig. S5c). In the image, you can distinguish between single capillary vessels (1), veins (2) and arteries (3). As shown in Fig. 7b, in a depth map image it is possible to identify capillaries surrounding the large veins and arteries through the height difference of the respective blood vessels. By increasing the voltage even further 3 × 4 mm, a wider range of images could be obtained, but the MEMS scanner would be susceptible to thermal damage. Even if a higher voltage could be constantly applied without damage, there are physical limits to the range of angular motion. As an alternative to increasing the moving angle of the scanner, we tried using an additional motorized stage to achieve a wider FOV. We obtained eight images under the same conditions as for the previous 2 mm × 2 mm image, and then superimposed the boundary regions of each image to convert them into a single image. The FOV of the completed PA image was 10 mm × 2 mm on the x and y axes, respectively (Fig. 7c). Similar to the previous image, arteries, veins, and microvasculature in the mouse ear are delineated. However, there is discontinuity at the boundaries between images, due to lags which manifest at the edges of the image. This lagging is an inherent MEMS scanner disadvantage, but it can be compensated for by applying additional feedback to the control signals, as mentioned earlier. Another possible source of image discontinuity is sample movement between frames. During imaging, the motor continues to move and pause, exerting forces that cause sample movement between frames. A B-scan cross-sectional image of the microvascular structure in the mouse ear was obtained to illustrate the discontinuity in the combined image (Fig. 7d). White triangles indicate discrete points with different depth levels between adjacent images. In the B-scan image, we can identify that some of the blood vessels are affected by echo artifacts caused by discontinuous interfaces. These echo artifact signals caused by reflections are weak compared to the original signals. On the contrary, in the depth-map image based on MAP (Fig. 7b), MAP picks up the highest signal in A-line, and the height information is directly translated from these highest signals. Thus, the echo artifacts shown in the cross-sectional image (Fig. 7d) does not significantly affect to the depth-map image processed by MAP.

### *In vivo* PA image of a human mole

**Figure 8:**
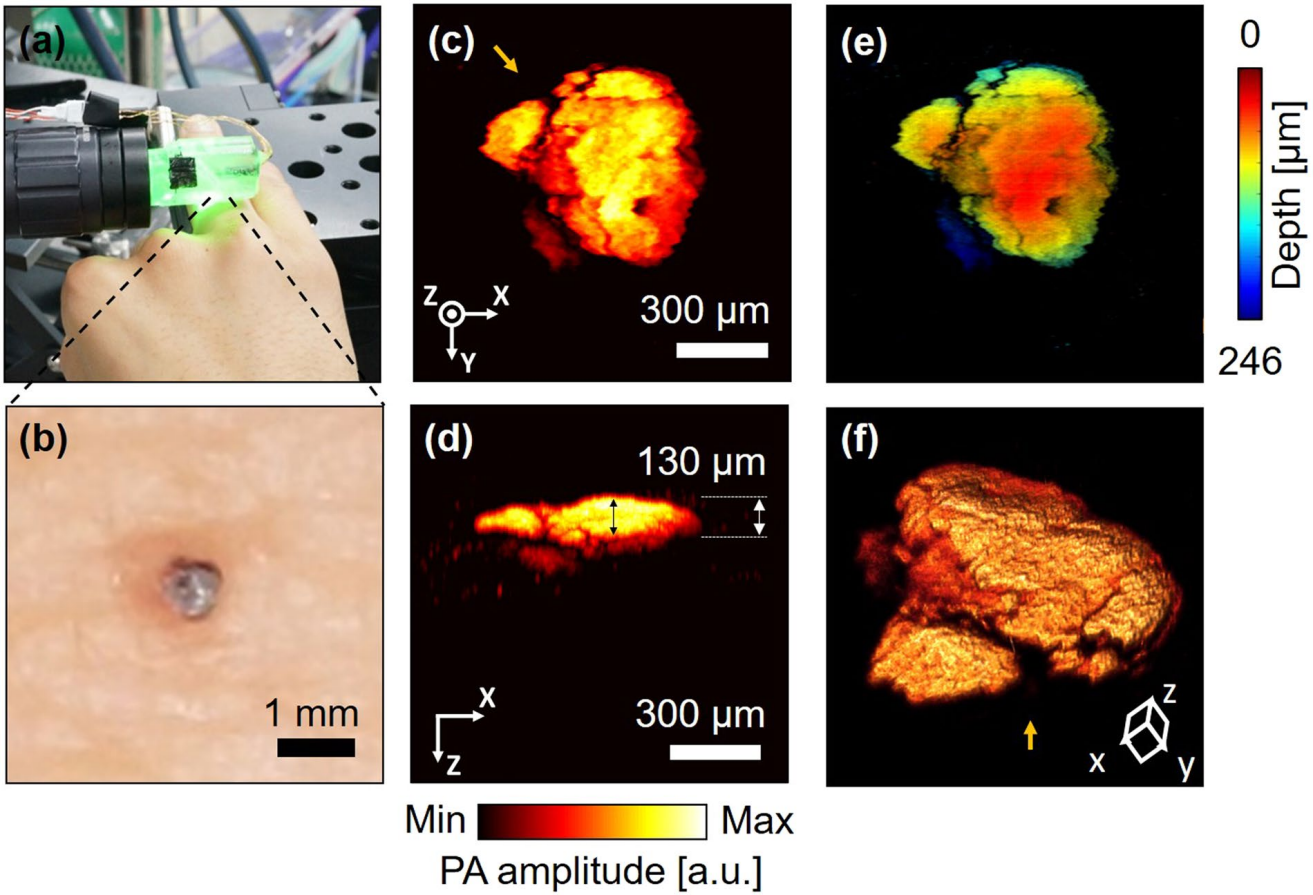
*In vivo* image of a mole on a human subject’s finger. (**a**) A photograph of the handheld PAM probe imaging the mole. (**b**) A photograph of the mole. (**c**) PA MAP image of the mole. (**d**) PA MAP image of the mole in cross-section. (**e**) Depth map image corresponding to PA MAP image (**c**); pseudo color encodes depth information. (**f**) 3D structure of the mole. Yellow arrow indicates the viewing direction corresponding to the yellow arrow direction in (**c**).

To demonstrate the system’s possible clinical application for melanoma discovery and boundary detection, we visualized a mole on a human subject’s finger (Fig. 8a). The optical image of the mole’s shape (Fig. 8b) matches with the PA MAP image (Fig. 8c). For clinical purposes, it is important to define the boundary and depth of moles. In this study, the depth profile of the mole was identified by a cross-sectional projection PA image (Fig. 8d). The estimated average thickness of the mole was 85 μm, and the thickest part was about 130 μm. In the depth map that was converted from depth values to colors, clear boundaries and the depth at each point are visible (Fig. 8e). Figure 8f shows the three-dimensional structure of the mole. The viewing direction is indicated by the yellow arrow in Fig. 8c.

## Discussion

We developed high SNR and high-speed handheld PAM probes using a two-axis waterproof microelectromechanical systems (MEMS) scanner for OR-PAM. All acoustic, mechanical, and optical components were integrated into a compact, easily employed single probe, 17 mm in diameter and weighing just 162 g. Using the numerical analysis, we figured out the conventional flat-tip electromagnet was not suitable for the cylinder, therefore changed it to the oblique-tip electromagnet. The position electromagnets were also optimized, and the movable front structure has been modified for the cylindrical structure. It was not easy to maintain the high resolution, the SNR, and the imaging rate while making the size of the handheld system small and movable. We demonstrated phantom images of various samples, including carbon fibers and electrospun microfibers. The system’s SNR, speed, FOV, resolution, and imaging depth were tested in phantom experiments. The measured lateral and axial resolutions were 12 μm ± 1.6 and 30 μm ± 3.1, respectively. At 532 nm, the measured SNR for a 6 μm carbon fiber was 39.2 dB, similar to the SNR of G2-OR-PAM. To obtain one PA image of 700 × 700 pixels, the measured B-scan and volumetric imaging rates were respectively 35 Hz and 0.05 Hz. The laser was operated at a repetition rate of 5 kHz to 50 kHz. The maximum FOV was 2.8 mm × 2 mm, with an applied voltage of 3 V on the x axis and 2 V on the y-axis, and the FOV could be extended using a motorized stage. Finally, *in vivo* images of a mouse’s ears, iris, and brain were collected, showing both a single capillary vessel and an artery and vein pair in a MAP image. We also imaged a human’s moles.

The most promising area for improvement is the imaging speed, which is most simply increased by using a laser with a faster repetition rate. The speed of sound in biological tissue is a known value, 1540 m/s. Assuming a maximum imaging depth of 1 mm, the time for ultrasound to travel from the maximum depth to the tissue surface is 0.65 μs. Therefore, the maximum effective pulse repetition rate of the laser is 1.54 MHz. If the laser pulse were faster than this repetition rate, the resulting ultrasound sound signal emitted from the tissue would overlap the previous PA signal. However, the value of 1.54 MHz is the hypothetical maximum value derived from the theoretical calculations, and other factors may affect the implementation of the laser. As the pulse repetition rate is increased the SNR would be decreased along with the power decrease. To compensate this, the power needs to be increased. Currently, other group imaged a mouse brain with the pulse repetition rate of 500 KHz laser and a picosecond pulse energy of 1 μJ with high SNR^37^. Nevertheless, simply employing a higher repetition rate laser does not guarantee faster imaging, unless the MEMS scanner physically supports the faster B-scan rate. By changing the width and the thickness of the torsional hinges of the scanner, we could modify their spring constant, optimizing the resonant frequency of the scanner. In addition, the 2A-WP-MEMS scanner does not draw an exactly linear raster scanning pattern. This slightly distorted pattern could be compensated for by applying an additional feedback mechanism to the control signals^38^.

Data acquisition and processing speed also argue for an increased pulse repetition rate. Currently the system stores and processes the image at 25 MB/s. If the laser pulse were 1.5 MHz, the program could store and process 0.75 GB of data per second. To deal with this tremendous data flow, a software system based on graphics processing units could be applied.

The closest medical application of this device is melanoma imaging and diagnosis. Melanoma can be cured almost immediately if they are diagnosed and treated in an early stage. However, as it progresses and spreads to other parts of the body, it causes the greatest number of deaths among skin cancer. By delineating the boundary and depth of human moles on the skin, this handheld OR-PAM system has demonstrated its ability to use melanoma detection and diagnostic tool.

## Materials and Methods

### Fabrication of the 2A-WP-MEMS scanner

The creation of this handheld PAM probe began with fabricating the 2A-WP-MEMS scanner. As a first step, we used an automated engraving machine (EGX-350, Roland, USA) to cut the rigid supporting pieces from a 1mm thick PMMA layer. To make the second layer from flexible PDMS, the layer pattern was engraved on an acrylic plate using a micromilling machine to make a mold. The base material and a curing agent (Sylgard 184 Silicone Elastomer Kit, Dow Corning, USA) were then mixed at a ratio of 10: 1, and the solution was stirred briskly for 10 minutes and degassed in a vacuum chamber for 30 minutes. Next, the prepared solution was placed in the mold and cured in a 60 °C oven for 12 hours to obtain the flexible layer. After complete curing, the flexible PDMS layer was peeled from the mold using a surgical blade and attached to the rigid PMMA support frame by plasma bonding. After the two layers were bonded, four neodymium magnets were inserted into cavities in the PDMS-PMMA structure. The aluminum mirror, attached to the center of the movable structure, was made by depositing an aluminum layer of 2000 Å thickness on a silicon wafer with a diameter of 4 inches and a thickness of 500 μm, using e-beam deposition. The wafer was then cut into 11 mm × 4 mm pieces using a wafer dicing machine. To fabricate the rear actuating structure, we fashioned four electromagnets by hand. The magnetic core is a soft iron nail, cut and polished at an angle of 45 degrees and annealed using a hot torch. Enameled copper wire with 50-μm diameter was wound around this iron core, and the measured resistance of the solenoid coil was 60 Ω. To control the movement of the MEMS scanner, these enameled wires were connected to the terminal connector of a high-current operational amplifier (OPA2544, Texas Instruments, USA). Through this connection, the actuation signal was sent to the scanner, the sawtooth signal was sent on the y-axis, and the sine signal was sent on the x axis. The entire rear actuating structure was sealed with PDMS for electrical isolation. Finally, the movable front structure was attached to the rear structure during the PDMS curing process.

### Numerical analysis and scanning responses of the 2A-WP-MEMS scanner

The conditions for numerical analysis of the magnetic field are explained in detail here. In the design, the gap between the solenoid coil and the magnet is 1.5 millimeter. The magnetic field value calculated by equation (1) was 3.2 mT. The calculated magnetic force $({\rm{F}}=\nabla ({\rm{m}}\cdot {\rm{B}}))$ on the iron core inside the solenoid coil was 0.4 mN. In COMSOL, we simulated a three-dimensional model assuming an atmospheric environment, ferromagnetic cores, and copper coils. To simulate multiple coil turns, we used a magnetic field mixing user interface and applied an AC current of 33 mA to multiple coil domains. The static electromotive forces applied on the angled and flat tip electromagnets were calculated using finite element method magnetic field (FEMM) software. The numerical results show that the angled tip (Fig. S1a) experiences 1.2 times greater static electromagnetic force perpendicular to its front layer than the flat tip does (Fig. S1b). To evaluate the performance of the scanner, we measured the range of the scanner’s mirror angle displacement in response to an applied voltage. When DC voltage was applied to the scanner, the mirror was held static, without rotation, so that the magnetic force between the electromagnet and the neodymium magnet could be compared. To determine the resonance frequency of the vibrating mirror, we applied 2 VAC and measured the maximum rotational angle that resulted, which was determined by the applied peak-to-peak amplitude and frequency. In the MEMS scanner, the maximum deflection angle of the mirror increased to 18 degrees along the x axis at 5 VAC and increased to 11 degrees along the y axis at 4 VAC. This angle provided a FOV of approximately 4 × 3 mm at a working distance of 7.3 mm from the mirror to the sample. However, for reliable operation of the system, most experiments were performed at 2 V, providing a 2 × 2 mm FOV.

### Configuration of the handheld PA microscopy probe

In our design, beams from a Q-switched diode-pumped solid-state laser (SPOT-10-200-532, Elforlight, UK) are coupled into a single-mode fiber (P1-460B-FC-1, Thorlabs, USA) for convenient light delivery. Two sets of mirrors (BB1-E02, Thorlabs, USA) and irises with a maximum aperture size of 2 mm (SM1D12D, Thorlabs, USA) are used to align the free-space beam path from the laser with a fiber collimator (TC25FC-543, Thorlabs, USA). The coupled beam is then aligned with the core axis of the fiber and transferred to the proximal end of the handheld PAM probe. There, the diverging output beam from the single-mode fiber enters a collimator (TC12FC-543, Thorlabs, USA) at the proximal end of the probe. The beam is focused by an objective lens (AC127-030-A, Thorlabs, USA) and passes through the OUC. In the middle of the OUC, silicone fluid (PMS-200, Dow Corning Corp, USA) is applied between two right-angle prisms (32–330, Edmund Optics) to transmit light but reflect ultrasound. The light beam passing through the OUC is reflected by the mirror surface on the MEMS scanner, then illuminates the target. The generated acoustic waves from the target are first reflected by the mirror surface on the MEMS scanner, and then by the silicon fluid plane in the middle of the OUC. Finally, the PA signals are detected by an ultrasound transducer (50 MHz center frequency, V214-BB-RM, Olympus NDT, USA). In our design, the aluminum mirror on the MEMS scanner reflects both light and ultrasound in water. To further maximize the SNR, an acoustic lens with a numerical aperture of 0.25 and a focal length of 11.6 mm (NT45-010, Edmund, USA) is mounted in front of the OUC. A command signal sent by a customized LabView program running on a PC is transferred to a function data acquisition (DAQ) board (NI PCIe-6321, National Instruments, USA) that generates a signal that triggers the laser. At the same time, this multi-functional DAQ initializes a digitizer (ATS9350-2G, Alazar Tech INC, Canada) to acquire acoustic signals via the ultrasound transducer. These signals are screened by a low pass filter (<80 MHz) and boosted by 56 dB using an amplifier (ZFL-500LN, Mini-Circuits, USA). At the same time, the DAQ controls the MEMS scanner, generating sine and sawtooth waves with specific frequencies to drive the scanner at different speeds. These sine and sawtooth signals are transferred to the scanner through a high-current operational amplifier (OPA2544, Texas Instruments, USA). The sawtooth wave signal controls the y axis of the scanner as the slow axis, and the sine wave signal controls the x axis of the scanner as the fast axis. The complicated housing structure was 3D printed using a transparent photo polymer (VeroClear-RGD810, Stratasys Ltd., USA). The complete handheld PAM probe is mounted on a portable 3-axis articulated arm (244 N, Manfrotto, Italy).

### Spatial resolutions and SNR of the handheld PAM probe

To measure the spatial resolutions of the system, we prepared a black and white resolution film mask for the lateral resolution and a carbon fiber for the axial resolution. The FOV of the obtained image was 0.27 mm × 0.36 mm along the x and y axes, and the applied voltage was 0.5 VAC on each axis. To obtain the lateral resolution, the PA profile data in the PA MAP image was extracted in the x direction along the line a-a’ (Fig. 4a). This normalized PA profile was converted to an edge spread function (ESF), which was then differentiated into a line spread function (LSF). The FWHM was obtained from this Gaussian profile. To measure the axial resolution, the extracted PA signal was normalized to a maximum value of 1 and then fitted to the LSF. The FWHM was again obtained from this Gaussian profile.

### Imaging an electrospun membrane vessel-mimicking phantom

To prepare electrospun polycaprolactone (PCL) thin films, 8.8 wt% PCL solution (440752, Sigma-Aldrich, USA) was added to chloroform (496189, Sigma-Aldrich, USA). After the mixed solution was stirred for 6 hours, the polymer solution was placed in a syringe, and 15.6 kV was applied across a distance of 14 cm between the tip of the syringe and a collector electrode. A 7 μ$\ell$ volume of the solution was then ejected from the syringe at 1.5 μ$\ell$/min. This process was carried out in a constant temperature and humidity room with at 22.5 °C and 20% relative humidity. The resulting fibers were collected on a ground electrode. To prepare the membrane test piece, a 5 mm × 5 mm square was cut from the mesh sheet using a surgical blade. The square of PCL fibers was then dyed with black ink to increase light absorption during PA imaging, and the sample was packaged with PDMS to protect the thin and fragile fibers from direct contact with the PAM probe. These two post-treatments caused the individual fibers to thicken and stick together.

For PA imaging, 2 V was applied to the x and y axes. The FOV for each image was 2 mm × 2 mm at the given voltage. The laser pulse energy on the sample was 8.6 mJ/cm2, which is less than the American National Standards Institute (ANSI) safety limit of 20 mJ/cm2 at 532 nm. The B-scan and volumetric imaging speeds to obtain a PA image of 700 × 700 pixels were 35 Hz and 0.05 Hz. The PA MAP image was configured using Labview software, and a continuous image was displayed on the monitor. Supplementary Video S1 is a live example. The imaging acquisition and display time for 700 pixels × 700 pixels along the x and y axes was 20 seconds, and the FOV was 2 mm × 2 mm. All PA images were reconstructed by depth correction along the x and y axes (Fig. S6), and depth encoded PA images were compensated by reconstruction of relative depth encoded images.

### *In vivo* small animal imaging

All animal experimental procedures were performed in accordance with laboratory animal protocols approved by the Institutional Animal Care and Use Committee of the Pohang University of Science and Technology (POSTECH), and regulations of the National Institutes of Health Guide for the Care and Use of Laboratory Animals. For *in vivo* small animal imaging, we used the same experimental parameters (i.e., the voltages applied to the MEMS scanner, FOV, imaging speed, and laser pulse energy) as those used in the phantom experiments. For small animal imaging, we prepared a normal white Balb/c mouse (3 weeks, 18 g). To image the blood vessels in the targeted area, the mouse was initially anesthetized by injecting 3% vaporized isoflurane gas into its air supply for 5 min. Anesthesia was checked by monitoring the response to pressing the tail of the mouse. After verifying anesthesia, we lowered the concentration of the gas to 0.75% during the remainder of the experiment. At that time, the mouse was placed on a heating pad to maintain a constant body temperature of 37 °C. To image the blood vessels in the mouse ear, the fur on the skin was shaved using an electronic trimmer, then depilated using a typical hair removal cream (Veet, Reckitt Benckiser LLC, USA). The nude ear was placed on an ear support bar to minimize interference from respiration-induced movement. In addition, acoustic gel was applied on the skin as an acoustic impedance matching material. Intermittently, eye drops were also applied to prevent dry eye and redness from a long experiment. While the mouse was unconscious, the iris pupil became dilated, so additional eye drops to induce pupil contraction were instilled in the eye to maintain the initial size of the pupil. To image the cortical vessels in the brain, the hair on the head was removed as before. A customized head holder fixed the mouse head on both sides at the ears. Then, the scalp was removed using a surgical blade and forceps, while the skull remained intact. The periosteum was also gently removed by cutting it away with surgical scissors. This step was conducted carefully to minimize bleeding.

### Enlarging the FOV

To obtain a large FOV, the applied voltage of the MEMS scanner was increased from 3 V in the x axis to 2 V in the y-axis. The achieved FOV was 4.1 × 2.9 mm, with 700 × 700 pixels along the x and y axes. In addition, we used a power stage (PT1-Z8, Thorlabs, USA) to obtain a large FOV image of the mouse ear by combining eight frames into one image. The FOV for each image was 2 mm × 2 mm in the x and y directions, and the image capture time was 20 seconds to get one frame of 700 × 700 pixels, just like any other image.

### *In vivo* human mole imaging

All human experimental procedures were performed in accordance with a protocol approved by the Institutional Review Board (IRB) of POSTECH. The volunteers were recruited from outside the lab to meet the requirement of the IRB regulation. Under the full explanation of the experiment, we received consent from all volunteers before the experiment. The volunteers fixed their fingers on the imaging stage while the handheld PAM probe scanned the imaging area, and we obtained volumetric image of the human mole. The applied laser power was 4.33 mJ/cm^2,^ which is less than ANSI safety limit. For Fig. 8e, the imaging process proceeded up to step 2, depth correction.

### Image processing

Typically, a PA image is obtained through the MAP process. You then calculate the index of each pixel in the MAP image to obtain the depth information (Fig. S46a). In conventional linear motor scanning, if there was no depth change of the scanning profile while the motor was moving, there is no problem, even if the MAP image is directly converted to the depth. However, in the case of a MEMS scanner, the scanning profile draws a parabola (Fig. S6b) because the scanner moves like a pendulum about its central axis. Therefore, for images obtained through a MEMS scanner, the depth of the scanning profile changes as the MEMS scanner moves, so the depth-encoded image needs to be corrected with the scanning profile of the MEMS scanner. By comparing the physical characteristics of the MEMS scanner with the images obtained, we are able to calculate the profile of the MEMS scanner. The x-axis motion is driven by a sine wave, and the Y-axis is driven by a sawtooth wave, so the profile of each axis shows different shapes, as described in Eq. 2 for the x axis and Eq. 3 for the y axis.

2 $${r}_{x}=\frac{{h}_{x}}{\cos [{\alpha }_{x}\times \,\cos (2\pi {F}_{c}t\,-\phi )+{\alpha }_{x}]}$$

3 $${r}_{y}=\frac{{h}_{y}}{\cos [-\frac{{\alpha }_{y}}{{T}_{c}}t+{\alpha }_{y}]}$$

where *r*_*x*_ and *r*_*y*_ are the corrected length from the mirror to the target sample along the respective x and y axes, *h*_*x*_ and *h*_*x*_ are the initial vertical length from the mirror to the target sample, *α*_*x*_ and *α*_*x*_ are the maximum moving angles on the respective x and y axes, *F*_*c*_ is a B-scan rate, *φ* is an initial phase on x axis, and *T*_*c*_ is a period for one volumetric imaging.

After correcting the depth information errors due to the MEMS scanner profile, we added a surface planarization process (Fig. S6c). Most images taken in this study are blood vessels near the skin surface of mice. To determine the relative height difference of each vessel, the uppermost blood vessel should be flat. However, since the skin of a living animal cannot always be leveled, the depth of the uppermost blood vessel was calculated first, and then the depth of each remaining vessel was calculated. For planarization of blood vessels, based on 3D position information, we obtained the plane of the PA signal from the strongest signal in each A-line. This plane was computed in MATLAB (R2016a, Mathworks, USA) using a 2D locally weighted scatterplot smoothing (LOWESS) fitting function. We then achieved planarization by subtracting the fitted plane of the subject from the final encoded depth image. In our calculations, because of the large computational load of 2D surface fittings, we used 1% of the position information to reduce computation. In addition, by using a Hessian matrix-based microvascular quantification (MQ) algorithm to quantify the density of electrospun microfibers, we segmented the respective threads in the MAP image. We separated the threads with a constant thickness using a thinning algorithm integrated in MATLAB, and measured the total length of the threads. Then, the density of the threads was calculated from Eq. 4 ^39^ as follows:

4 $$Thread\,density=\frac{Total\,thread\,length}{Selected\,area}\,[mm/m{m}^{2}]$$

To illustrate the image correction process, we present images of the electrospun microfibers (Fig. S7). In the depth-encoded images, the morphology of the sample in the image is different from the true shape of the sample. The center of the microfiber membrane protrudes, and the mouse ear is almost flat. The depth map image of the membrane was corrected by using equations 2 and 3 (Fig. S7c).

## Electronic supplementary material

 Supplementary Information

In vivo PA imaging movie of a mouse ear.

In vivo 3D volumetric imaging of the vasculature in a mouse ear.

## References

[1] Wang LHV, Hu S (2012). Photoacoustic Tomography: In Vivo Imaging from Organelles to Organs. Science.

[2] Hu S, Maslov K, Wang LV (2011). Second-generation optical-resolution photoacoustic microscopy with improved sensitivity and speed. Opt Lett.

[3] Yao J, Maslov K, Hu S, Wang LV (2009). Evans blue dye-enhanced capillary-resolution photoacoustic microscopy in vivo. J Biomed Opt.

[4] Wang LD, Maslov K, Wang LHV (2013). Single-cell label-free photoacoustic flowoxigraphy in vivo. P Natl Acad Sci USA.

[5] Hu S, Maslov K, Tsytsarev V, Wang LV (2009). Functional transcranial brain imaging by optical-resolution photoacoustic microscopy. J Biomed Opt.

[6] Yao J, Maslov KI, Shi Y, Taber LA, Wang LV (2010). *In vivo* photoacoustic imaging of transverse blood flow by using Doppler broadening of bandwidth. Opt Lett.

[7] Yao, J. J., Maslov, K. I., Zhang, Y., Xia, Y. N. & Wang, L. V. Label-free oxygen-metabolic photoacoustic microscopy *in vivo*. *Journal of Biomedical Optic*s **1**6, doi:Artn 07600310.1117/1.3594786 (2011). 10.1117/1.3594786 PMC3144973 21806264

[8] Hu S, Yan P, Maslov K, Lee JM, Wang LV (2009). Intravital imaging of amyloid plaques in a transgenic mouse model using optical-resolution photoacoustic microscopy. Opt Lett.

[9] Weber J, Beard PC, Bohndiek SE (2016). Contrast agents for molecular photoacoustic imaging. Nat Methods.

[10] Kim C (2010). *In vivo* molecular photoacoustic tomography of melanomas targeted by bioconjugated gold nanocages. ACS Nano.

[11] Homan KA (2012). Silver nanoplate contrast agents for in vivo molecular photoacoustic imaging. ACS Nano.

[12] Razansky D, Ntziachristos V (2007). Hybrid photoacoustic fluorescence molecular tomography using finite-element-based inversion. Med Phys.

[13] Ntziachristos V, Razansky D (2010). Molecular imaging by means of multispectral optoacoustic tomography (MSOT). Chem Rev.

[14] Razansky D (2009). Multispectral opto-acoustic tomography of deep-seated fluorescent proteins *in vivo*. Nature Photonics.

[15] Danielli A (2014). Label-free photoacoustic nanoscopy. J Biomed Opt.

[16] Galanzha EI, Shashkov EV, Spring PM, Suen JY, Zharov VP (2009). *In vivo*, noninvasive, label-free detection and eradication of circulating metastatic melanoma cells using two-color photoacoustic flow cytometry with a diode laser. Cancer Res.

[17] He G, Xu D, Qin H, Yang SH, Xing D (2015). *In vivo* cell characteristic extraction and identification by photoacoustic flow cytography. Biomed Opt Express.

[18] Lin R (2015). Longitudinal label-free optical-resolution photoacoustic microscopy of tumor angiogenesis in vivo. Quant Imaging Med Surg.

[19] Yao DK, Maslov K, Shung KK, Zhou Q, Wang LV (2010). *In vivo* label-free photoacoustic microscopy of cell nuclei by excitation of DNA and RNA. Opt Lett.

[20] Strohm EM, Berndl ESL, Kolios MC (2013). Probing Red Blood Cell Morphology Using High-Frequency Photoacoustics. Biophys J.

[21] Strohm, E. M., Moore, M. J. & Kolios, M. C. Single Cell PhotoacousticMicroscopy: A Review. *Ieee J Sel Top Quant* **22**, doi:Artn 680121510.1109/Jstqe.2015.2497323 (2016).

[22] Bost, W*. et al*. High Frequency Optoacoustic Microscopy. *Ieee Eng Med Bio*, 5883–5886, doi:10.1109/Iembs.2009.5334452 (2009). 10.1109/IEMBS.2009.5334452 19964880

[23] Strohm EM, Berndl ES, Kolios MC (2013). High frequency label-free photoacoustic microscopy of single cells. Photoacoustics.

[24] Silverman RH (2010). High-Resolution Photoacoustic Imaging of Ocular Tissues. Ultrasound in Medicine and Biology.

[25] Oladipupo SS (2011). Conditional HIF-1 induction produces multistage neovascularization with stage-specific sensitivity to VEGFR inhibitors and myeloid cell independence. Blood.

[26] Oladipupo S (2011). VEGF is essential for hypoxia-inducible factor-mediated neovascularization but dispensable for endothelial sprouting. Proc Natl Acad Sci USA.

[27] Hsu HC, Wang L, Wang LV (2016). *In vivo* photoacoustic microscopy of human cuticle microvasculature with single-cell resolution. J Biomed Opt.

[28] Hu, S., Maslov, K. & Wang, L. V. Three-dimensional Optical-resolution PhotoacousticMicroscopy. *Jove-J Vis Ex*p, doi:ARTN e272910.3791/2729 (2011). 10.3791/2729 PMC3197122 21587156

[29] Hajireza P, Shi W, Zemp RJ (2011). Real-time handheld optical-resolution photoacoustic microscopy. Opt Express.

[30] Zafar H, Breathnach A, Subhash HM, Leahy MJ (2015). Linear-array-based photoacoustic imaging of human microcirculation with a range of high frequency transducer probes. J Biomed Opt.

[31] Needles A (2013). Development and initial application of a fully integrated photoacoustic micro-ultrasound system. IEEE Trans Ultrason Ferroelectr Freq Control.

[32] Needles A (2010). Nonlinear contrast imaging with an array-based micro-ultrasound system. Ultrasound Med Biol.

[33] Kim JY, Lee C, Park K, Lim G, Kim C (2015). Fast optical-resolution photoacoustic microscopy using a 2-axis water-proofing MEMS scanner. Sci Rep.

[34] Kim JY, Lee C, Park K, Lim G, Kim C (2015). A PDMS-Based 2-Axis Waterproof Scanner for PhotoacousticMicroscopy. Sensors-Basel.

[35] Siegel RL, Miller KD, Jemal A (2017). Cancer statistics, 2017. CA: A Cancer Journal for Clinicians.

[36] Duvernoy HM, Delon S, Vannson JL (1981). Cortical blood vessels of the human brain. Brain Res Bull.

[37] Yao, J. J. *et al*. High-speed label-free functional photoacoustic microscopy of mouse brain in action. *Nat Methods* **12**, 407−+, 10.1038/nmeth.3336 (2015). 10.1038/nmeth.3336 PMC4428901 25822799

[38] Yoo HW, Ito S, Schitter G (2016). High speed laser scanning microscopy by iterative learning control of a galvanometer scanner. Control Eng Pract.

[39] Yang ZY (2014). Multi-parametric quantitative microvascular imaging with optical-resolution photoacoustic microscopy *in vivo*. Opt Express.

