# Supplementary material for: Handheld Photoacoustic Microscopy Probe
Source: Sci Rep. 2017 Oct 17;7:13359. doi: 10.1038/s41598-017-13224-3 (PMC5645466; doi:10.1038/s41598-017-13224-3)
Supplement: Supplementary file 1 — Supplementary Information [file 41598_2017_13224_MOESM1_ESM.doc]

Supplementary information

**Handheld Photoacoustic Microscopy Probe**

**Kyungjin Park1†, Jin Young Kim2†, Changho Lee2**¶**, Seungwan Jeon2, Geunbae Lim1,3* and Chulhong Kim1, 2, 3***

1School of Interdisciplinary Bioscience and Bioengineering, Pohang University of Science and Technology (POSTECH), 77 Cheongam-ro, Nam-gu, Pohang, Gyeongbuk, Republic of Korea, 37673

2Department of Creative IT Engineering, Pohang University of Science and Technology (POSTECH), 77 Cheongam-ro, Nam-gu, Pohang, Gyeongbuk, Republic of Korea, 37673

3Department of Mechanical Engineering, Pohang University of Science and Technology (POSTECH), 77 Cheongam-ro, Nam-gu, Pohang, Gyeongbuk, Republic of Korea, 37673

†These authors contributed equally to this work

¶ Current address: Department of Nuclear medicine, Chonnam National University Medical School & Hwasun Hospital, 160 Baekseo-ro, Gwangju, 61469, South Korea

*Correspondence and requests for materials should be addressed to C.K. ([chulhong@postech.edu](mailto:chulhong@postech.edu))

**Fig. S1**

**
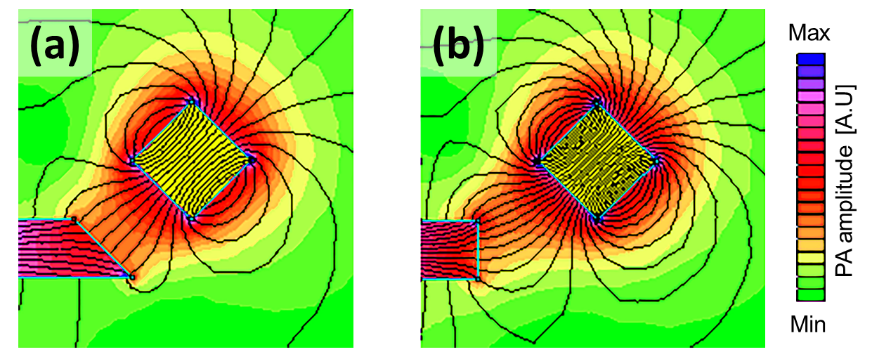
**

**Supplementary Figure. S1.** Numerical analysis of the magnetic field between the ND magnets and electromagnets, using finite element method magnetic software (FEMM). Representations of the magnetic fields from (a) an oblique-tip electromagnet and (b) a flat-tip electromagnet oriented toward the ND magnets.

**Fig. S2**

**
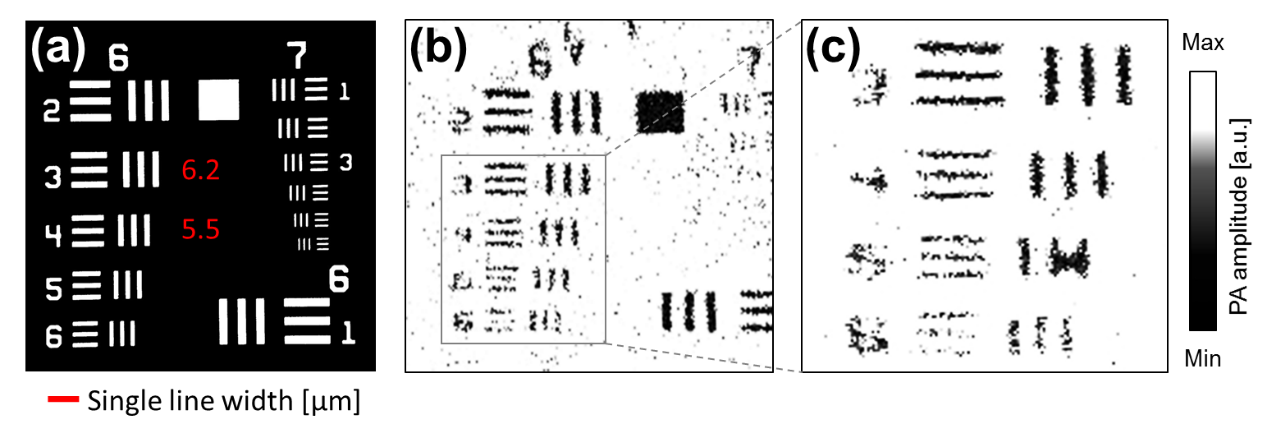
**

**Supplementary Figure. S2.** PA MAP images of the USAF resolution test chart. (a) Pattern of group 6 and 7 in the USAF resolution test chart (b) PA MAP image of group 6 in the USAF resolution test chart (c) PA MAP image of row 3, 4, 5, and 6 in the group 6. Each line width of the rows are 6.2, 5.5, 4.9, and 4.4 μm respectively.

**Fig. S3**


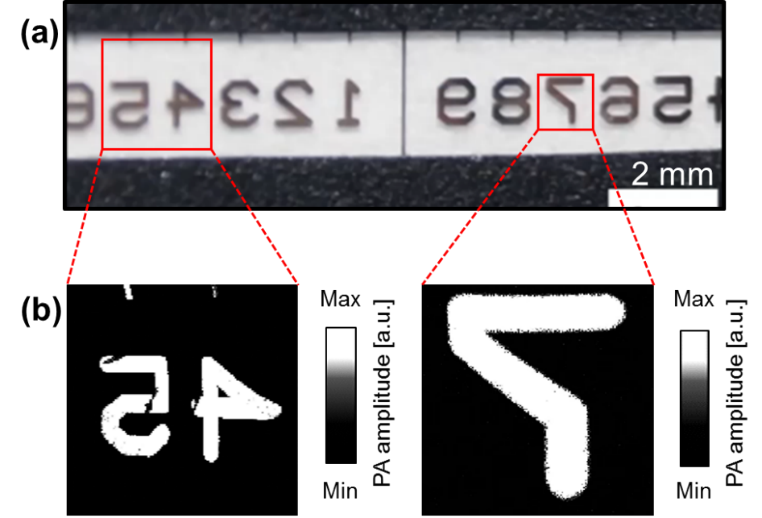


**Supplementary Figure. S3.** PA images of numbers on a micro-ruler (a) a picture of micro-ruler (b) PA MAP images of number 4 and 5 (FOV 2 mm by 2mm), and PA MAP image of number 7 (FOV 1 mm by 1mm) on the micro-ruler.

**Fig. S4**

**
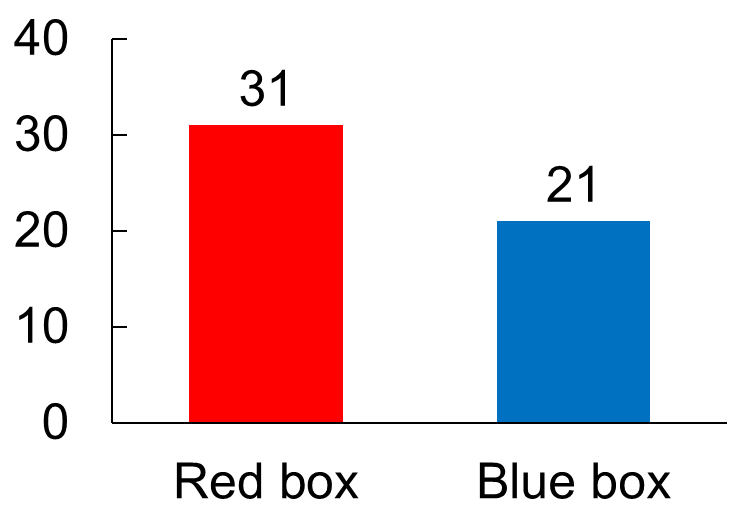
**

**Supplementary Figure. S4.** The calculated total length of the fibers in the designated boxes. The blue box encloses an area near the center, and the red box indicates an area near the boundary of the electro spun microfibers. The calculated total length of the fibers in the blue box was 21 mm, and the total fiber length in the red box was 31 mm.

**Fig. S5**

**
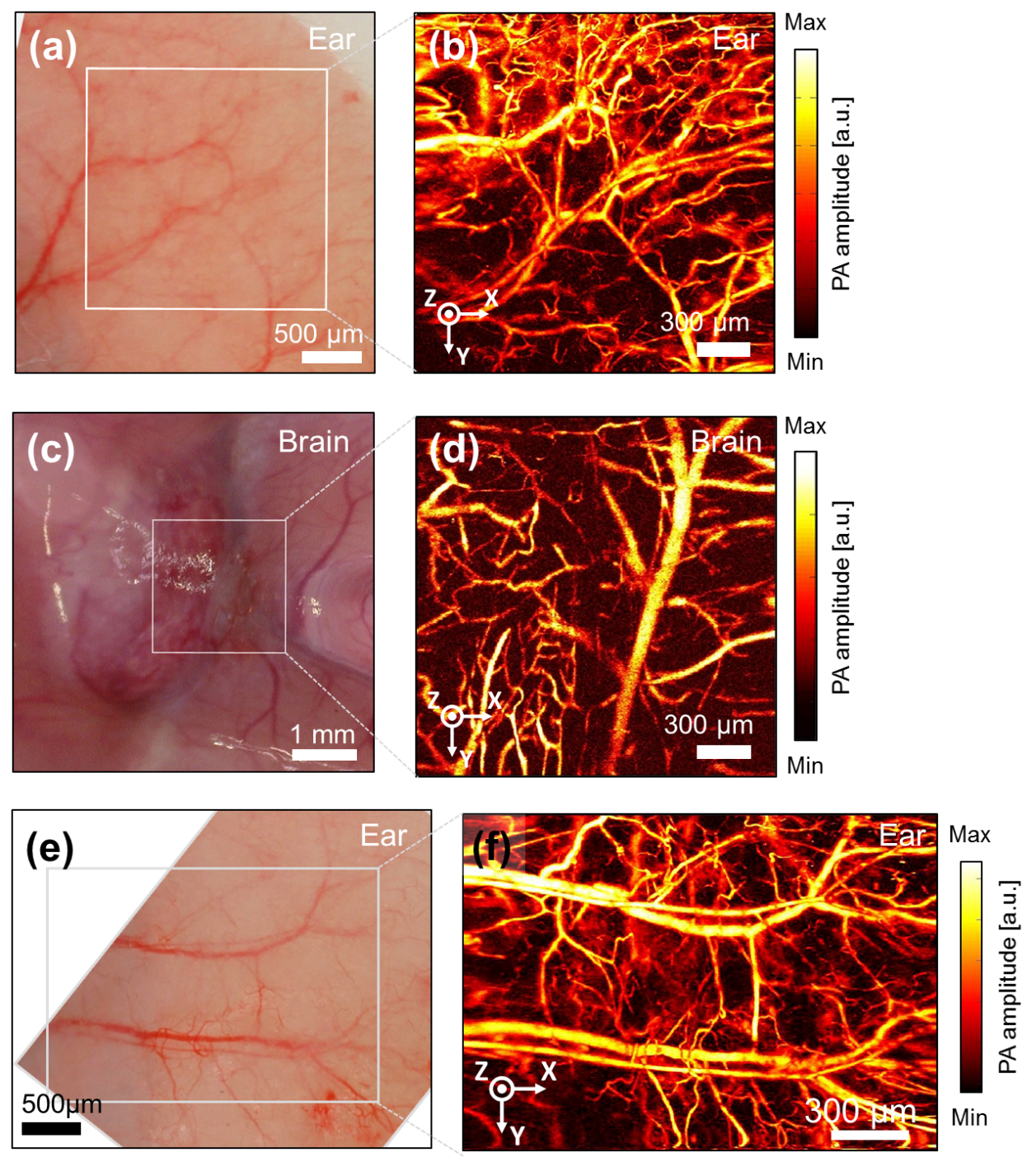
**

**Supplementary Figure. S5.** Pictures of microvasculature in a mouse ear and brain (a), (c), (e) Pictures of microvasculature in the mouse ear and brain. (d), (e), (f) PA MAP images of microvasculature in the mouse ear and brain which are corresponds to the pictures.

**Fig. S6**

**
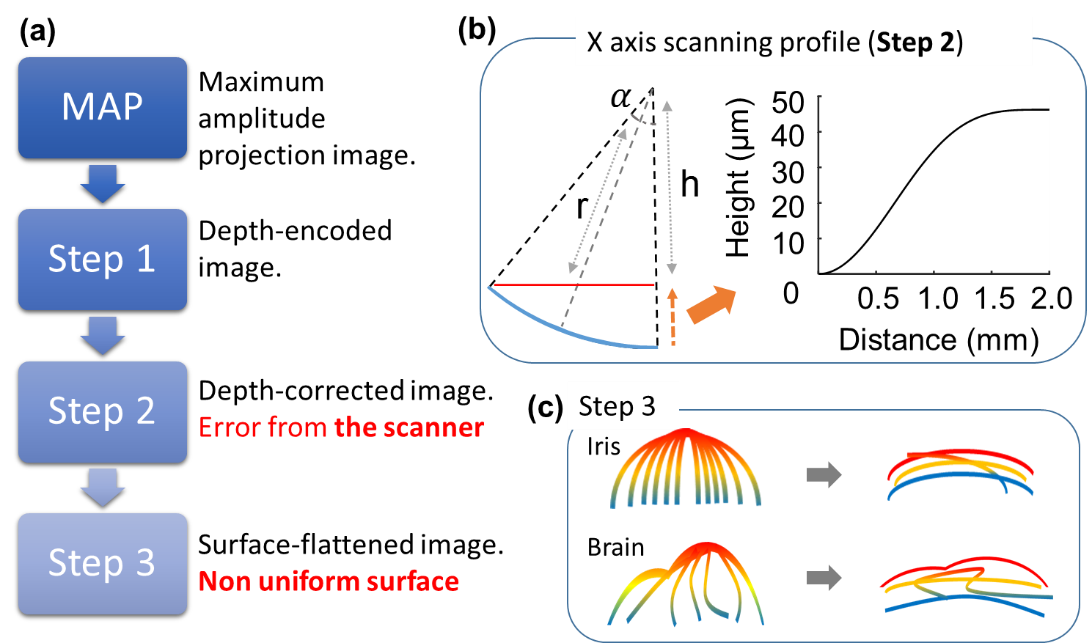
**

**Supplementary Figure. S6.** Depth map image process. (a) Block diagram of the entire correction process. (b) Description of the depth-correction process; Blue line indicates the scanning profile and red line indicates the corrected depth profile. (c) Simplified diagram of the surface-flattening process. MAP, maximum amplitude projection.

**Fig. S7**

**
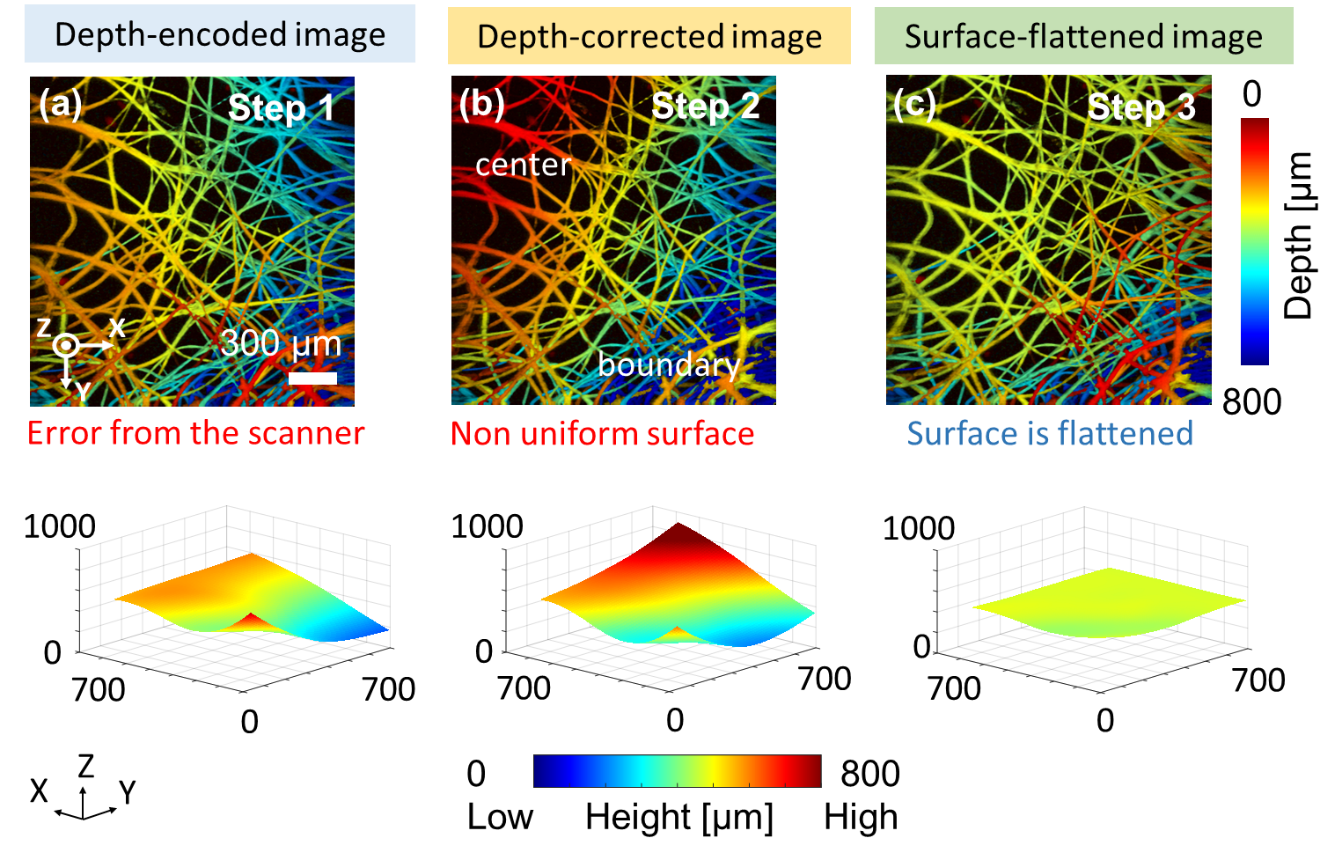
**

**Supplementary Figure. S7.** Examples of the depth mapping process. (a) Depth-encoded image of the electrospun microfibers and the surface profile extracted via 2D LOWESS fitting from the depth-encoded image. (b) Depth-corrected image of the electrospun microfibers and the surface profile. (c) Surface-flattened image of the electrospun microfibers and the surface profile. LOWESS, locally weighted scatterplot smoothing.

**Supplementary Video. S1**.


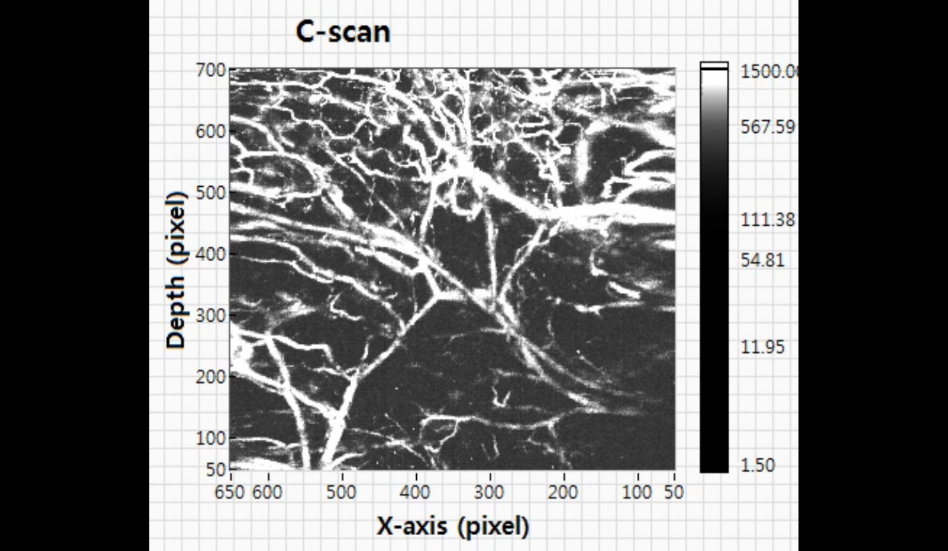


**Supplementary Video. S1**. *In vivo* PA imaging movie of a mouse ear. 700 B-scan images and one corresponding PA MAP image are displayed in 20 seconds.

**Supplementary Video. S2.**


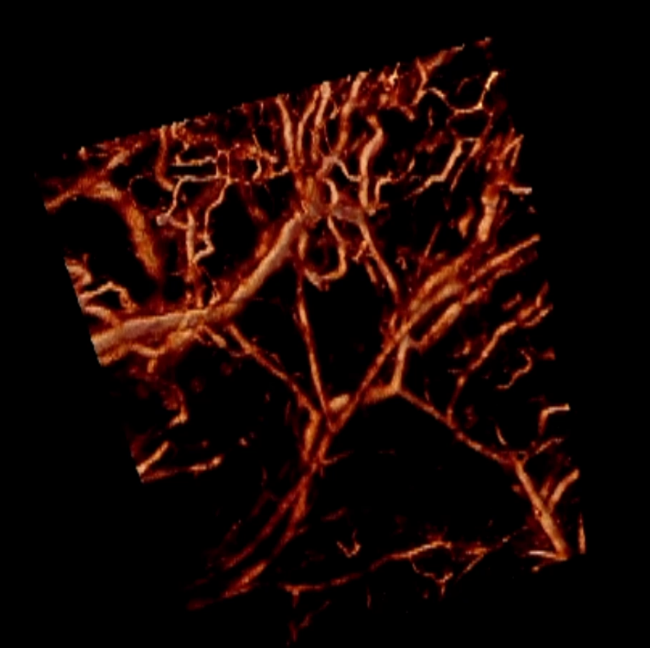


**Supplementary Video. S2**. *In vivo* 3D volumetric imaging of the vasculature in a mouse ear. The movie was processed by commercial software (Amira 6, FEI, USA).
